# The oncogenic role of SAMMSON lncRNA in tumorigenesis: A comprehensive review with especial focus on melanoma

**DOI:** 10.1111/jcmm.17978

**Published:** 2023-09-29

**Authors:** Majid Ghasemian, Hossein Babaahmadi‐Rezaei, Azam Khedri, Chandrabose Selvaraj

**Affiliations:** ^1^ Department of Clinical Biochemistry, Faculty of Medicine Ahvaz Jundishapur University of Medical Sciences Ahvaz Iran; ^2^ Center for Transdisciplinary Research, Department of Pharmacology, Saveetha Dental College and Hospitals Saveetha Institute of Medical and Technical Sciences (SIMATS), Saveetha University Chennai Tamil Nadu India

**Keywords:** cancer, epigenetic, LncRNA, SAMMSON, signalling pathway

## Abstract

LncRNA Survival Associated Mitochondrial Melanoma Specific Oncogenic Non‐coding RNA (SAMMSON) is located on human chromosome 3p13, and its expression is upregulated in several tumours, including melanoma, breast cancer, glioblastoma and liver cancer and has an oncogenic role in malignancy disorders. It has been reported that SAMMSON impacts metabolic regulation, cell proliferation, apoptosis, EMT, drug resistance, invasion and migration. Also, SAMMSON is involved in regulating several pathways such as Wnt, MAPK, PI3K, Akt, ERK and p53. SAMMSON is considered a potential diagnostic and prognostic biomarker in several types of cancer and a suitable therapeutic target. In addition, the highly expressed SAMMSON is closely associated with clinicopathological features of various cancers. SAMMSON has a significant role in regulating epigenetic processes by regulating histone protein or the status of DNA methylation. Herein for the first time, we comprehensively summarized the currently available SAMMSON, molecular regulatory pathways, and clinical significance. We believe that clarifying all the molecular aspects of this lncRNA can be a good guide for cancer studies in the future.

## 1 INTRODUCTION

In recent years, researchers using RNA sequencing (RNA‐seq) technologies revealed that approximately 98% of human genome transcribed do not translate to any proteins, and these transcripts are categorized as non‐coding RNAs (ncRNAs).^1^, ^2^ A subclass of ncRNAs that have over ~200 nt in length is classified as long non‐coding RNAs (lncRNAs).^3^, ^4^ According to NONCODEV5 dataset in the human genome over 100,000 lncRNA exist.^5^ Numerous studies have shown that lncRNAs regulate many cellular processes, including proliferation, differentiation, cell cycle, apoptosis, drug resistance, epigenetic modifications, RNA/protein stability, transcription, translation and post‐transcriptional modifications.^6^, ^7^, ^8^ LncRNA Survival Associated Mitochondrial Melanoma Specific Oncogenic Non‐coding RNA (SAMMSON), also known as LINC01212, has been identified by Leucci and colleagues.^9^ SAMMSON is located on the 3p13 chromosome and exists 4 exons and 28 transcripts that are produced through alternative splicing (https://asia.ensembl.org/Homo_sapiens/Gene/Splice?db=core;g=ENSG00000240405;r=3:69999550‐70518064). Upregulation of SAMMSON was observed in several cancers, including melanoma,^10^ breast cancer,^11^ glioblastoma^12^ and liver cancer^13^; also, SAMMSON played a critical role in intracranial aneurysms.^14^ Past studies reveal that approximately 10% of melanoma patients involved focal amplifications of chromosome 3p13–3p14. Several oncogenes such as melanocyte inducing transcription factor (MITF) and SAMMSON exist in this amplicon that has a pivotal function in melanoma genesis. MITF resided approximately ~30 kb upstream of SAMMSON and has consistently co‐gained with SAMMSON. However, the transcription factor SOX10 targets SAMMSON. SOX10 is a melanoblast/melanoma‐specific transcription factor with a binding site upstream of SAMMSON, and its silencing decreases SAMMSON expression.^9^, ^10^ This literature review, for the first time, comprehensively summarizes the research progress of SAMMSON, molecular regulatory pathways, and clinical significance. This will help to clarify SAMMSON crucial role and offer guidance for SAMMSON studies in the future.

## 2 SAMMSON AND TUMORIGENESIS

Previous studies have shown that the expression level of SAMMSON was upregulated in several malignancies. The overexpression of SAMMSON can promote tumour progression by increasing cell proliferation, invasion, migration, epithelial‐mesenchymal transition (EMT), chemoresistance, and apoptosis suppression. Also, SAMMSON plays a crucial role in tumour growth by affecting different aspects of mitochondrial metabolism. Past studies reported that SAMMSON affects on different signalling pathways such as Wnt, MAPK, PI3K, Akt, ERK and p53 and alters cancerous cells behaviour. In the following, the role of SAMMSON in various cancers is examined in more detail.

### 2.1 SAMMSON effects on different aspects of melanoma

**FIGURE 1:**
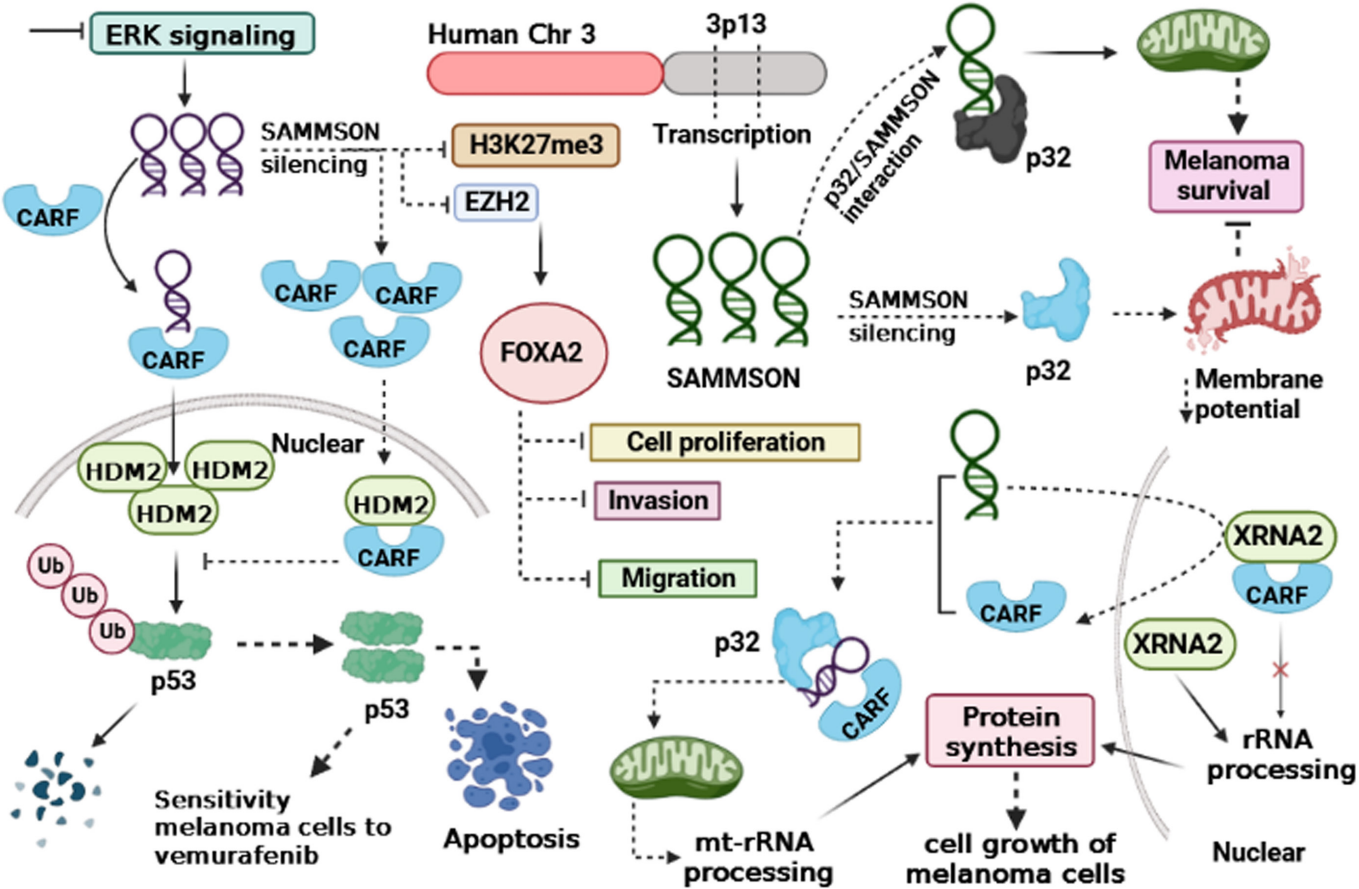
SAMMSON via different mechanisms exerts its oncogenic roles in melanoma. SAMMSON promotes melanoma progression by affecting different signalling pathways, mitochondria biogenesis, protein synthesis, cell proliferation, invasion and migration. The overexpression of SAMMSON inhibits the localisation of CARF to nucleus which leads ubiquitination of p53 by HDM2 and follows its degradation. After silencing SAMMSON, CARF can translocate to the nucleus and interacts with HDM2, which inhibits ubiquitination of p53. On the contrary, silencing of SAMMSON increases FOXA2 expression that inhibits tumour progression. SAMMSON via interaction with CARF and p32 plays a pivotal role in the processing of mt‐rRNA and protein synthesis. Ub: Ubiquitin, CARF: Collaborator of Rapidly accelerated fibrosarcoma, HDM2 (or MDM2): Is an E3 ubiquitin‐protein ligase, EZH2: Enhancer of zeste homologue 2, H3K27me3: Trimethylation of lysine 27 in histone H3, FOXA2: Forkhead box protein A2, XRN2: 5′‐3′ exoribonuclease 2, Mt: Mitochondria, Chr: Chromosome.

Several studies have shown that SAMMSON expression is increased in tissue and cancerous cells of melanoma.^9^, ^10^, ^15^, ^16^, ^17^ According to the literature, p32 expression is elevated in tumours. Similarly, p32 plays a crucial role in developing mitochondrial 16S rRNA and is involved in mitochondrial metabolism through modulation of its protein synthesis. On the contrary, p32 is responsible for maintaining mitochondrial integrity and homeostasis.^18^, ^19^, ^20^, ^21^ Melanoma cells were significantly less clonogenic, resistant to MAPK inhibitors and invasive after SAMMSON silencing. SAMMSON can localize into the cytoplasm with a fraction co‐localising with 16 s rRNA. SAMMSON, through interaction with p32 can enhance the localisation of mitochondrial and its function. After the silencing of SAMMSON, the membrane potential of mitochondrial remarkably reduced. Taken together, silencing of SAMMSON decreases melanoma survival, and it is clear that SAMMSON exerts this function by modulating SAMMSON/p32/ mitochondrial axis (Figure 1; Table 1).^9^ Rapidly accelerated fibrosarcoma (RAF) proteins are a family of Ser/Thr kinases and have three members known as CRAF, BRAF and ARAF that can activate the MAP kinase/ERK‐signalling pathway. BRAF mutations are common in malignant melanomas.^28^, ^29^, ^30^ Han et al. reported that upon ERK pathway inhibition, the expression of SAMMSON remarkably increased. According to their findings, BRAF inhibitor vemurafenib resistance is augmented by SAMMSON overexpression. Also, SAMMSON can increase p53 degradation by interaction with CARF. On the contrary, after silencing, the SAMMSON's, p53 pathway is activated via inhibition of its proteasomal degradation. Also, after the depletion of SAMMSON, the sensitivity of mutant BRAF melanoma cells to vemurafenib increased, and the rate of apoptosis cells via induction of cleavage of caspase‐3 and caspase‐9 was promoted. SAMMSON via, modulating CARF‐p53 signalling axis, plays a pivotal role in adaptive resistance to BRAF inhibitors.^10^ In uveal melanoma (UM) (the most common eye tumour in adults), the expression level of SAMMSON was upregulated in tissues and cancer cell lines. Shanna et al. showed that inhibition of SAMMSON suppressed cell growth and viability but induced cell apoptosis. The silencing of SAMMSON has effects on the process of protein synthesis and the function of mitochondria. SAMMSON exerts this function via interaction with p32, MRPL13 and XRN2. Mitochondrial respiration and mitochondrial capacity were decreased upon the knockdown of SAMMSON. Additionally, the inhibition of SAMMSON in through the inhibitor of MEK kinase (trametinib) results in a reduction in the number of viable cells and the growth of tumours.^15^, ^16^ Yang et al. reported that upon silencing of SAMMSON, cell proliferation, invasion, and migration were inhibited. However, after SAMMSON silencing, FOXA2 expression increased while methylated histone H3 (H3K27me3) and enhancer of zeste homologue 2 (EZH2) expression decreased. They also reveal that ectopic expression of FOXA2 remarkably reduces cell proliferation, invasion, migration and tumour growth in melanoma cells. It can be concluded that SAMMSON, by modulating EZH2/H3K27me3 axis, regulates FOXA2 expression. Subsequently, FOXA2 impacts different aspects of phenotype malignancy in melanoma.^17^ Malignant cancerous cells for providing metabolic demand are seriously dependent on protein synthesis. Roberto et al. reported that SAMMSON elevated rRNA maturation and protein synthesis in melanoma cancer cells. It exerts this function via modulation of the localisation of CARF. Both proteins XRNA2 and CARF have a critical role in the biogenesis of cellular ribosomes. In a normal state (without SAMMSON) CARF via interaction with XRNA2 form a complex in the nucleoplasm, but in the presence of SAMMSON (in melanoma cells) the interaction between CARF and p32 was facilitated, which led to disrupted interaction between CARF and XRNA2. Next, XRNA2 increases rRNA processing; conversely, CARF/p32 increases mitochondrial‐rRNA processing. It can be concluded that SAMMSON increases the cell growth of melanoma cells by promoting protein synthesis.^22^

**TABLE 1:** Summary of studies which assessed expression, function, and regulatory mechanisms of SAMMSON in different types of cancers

Cancer type	Clinical samples	Assessed cell lines	SAMMSON expression	Effects: in vitro and in vivo	Regulatory mechanism	Ref
Melanoma	67 malignant melanoma and 11 nevi tissues	SK‐MEL‐110, A375, A875, SK‐MEL‐28, and M21, ACHN, CaKi‐1, 786‐O, HaCaT	UP	▼ SAMMSON: ↓cell proliferation, ↓invasion, ↓migration	SAMMSON /EZH2/H3K27me3/ FOXA2	^17^
	‐	A375, HEK293FT, 1205Lu, A375 TR, 1205Lu TR, SK‐MEL‐28	UP	▼ SAMMSON: Activated p53 signalling, Sensitized melanoma cells to RAF inhibitor, ↑ rate of apoptosis	SAMMSON/CARF/p53	^10^
	‐	SK‐MEL‐28, SKMEL‐28, LCLs, Mel‐ST, HEK293T, NHEM, MM	UP	∆ SAMMSON: ↑ Cell growth, ↑ protein synthesis	SAMMSON/ CARF/XRN2/p32	^22^
Uveal melanoma	21 Paired tissues	92–1, OMM1, OMM2.3, MEL270, MP38, MP46, MM28, MP65, CRMM1, CRMM2, MEL077, SK‐MEL28, HEK293T, CT5.3hTERT	UP	▼ SAMMSON: ↓Growth, ↓Cell viability, ↑Apoptosis, ↓mitochondrial function	SAMMSON/p32	^15^
BC	68 TNBC Paired tissues	HCC70 and BT‐549	UP	∆ SAMMSON: ↑Cell proliferation	SAMMSON/p53	^23^
	‐	MCF‐7, MCF‐7dox	UP	▼ SAMMSON:↓Chemoresistance, ↓glycolytic metabolism, ↓ROS production	metabolic rewiring with improvement of oxidative metabolism	^11^
GC	126 Paired tissues	SGC‐7901, HCG‐27, AGS, MGC803	UP	▼ SAMMSON: ↓invasion, ↓migration	‐	^24^
HCC	70 Paired tissues	SNU‐182, SNU‐398	UP	∆ SAMMSON: ↑invasion, ↑migration	SAMMSON/miR‐9‐3p	^13^
	19 peri‐tumour, 7 early HCC and 12 aHCC samples	293 T	UP	∆ SAMMSON: ↑liver TIC self‐renewal, ↑tumour propagation, ↑invasion	SAMMSON/EZH2/ CTNNBIP1/ Wnt/β‐ catenin pathway	^25^
GBM	19 Paired tissues	U87MG, T98G, U251, LN229, A172, NHA	UP	▼ SAMMSON:	SAMMSON/PI3K/Akt	^12^
	56 GBM, and 35 control	U87, U‐373 MG	UP	∆ SAMMSON: ↑ cell proliferation	SAMMSON/ miR‐622	^26^
PTC	70 Paired tissues	PC‐1, HTH83, K1, 8505C, SW1736, BCPAP, Nthy‐ori 3–1	UP	▼ SAMMSON: ↓cell proliferation, ↓invasion, ↓tumorigenicity, ↓metastasis	SAMMSON/ p300/Sp1	^27^

Abbreviations: BC, breast cancer, GBM, glioblastoma, GC, gastric cancer, HCC, hepatocellular carcinoma, PTC, papillary thyroid carcinoma, ∆, over‐expression, ▼, knockdown, ↑, increase, ↓, decrease.

### 2.2 Breast cancer

**FIGURE 2:**
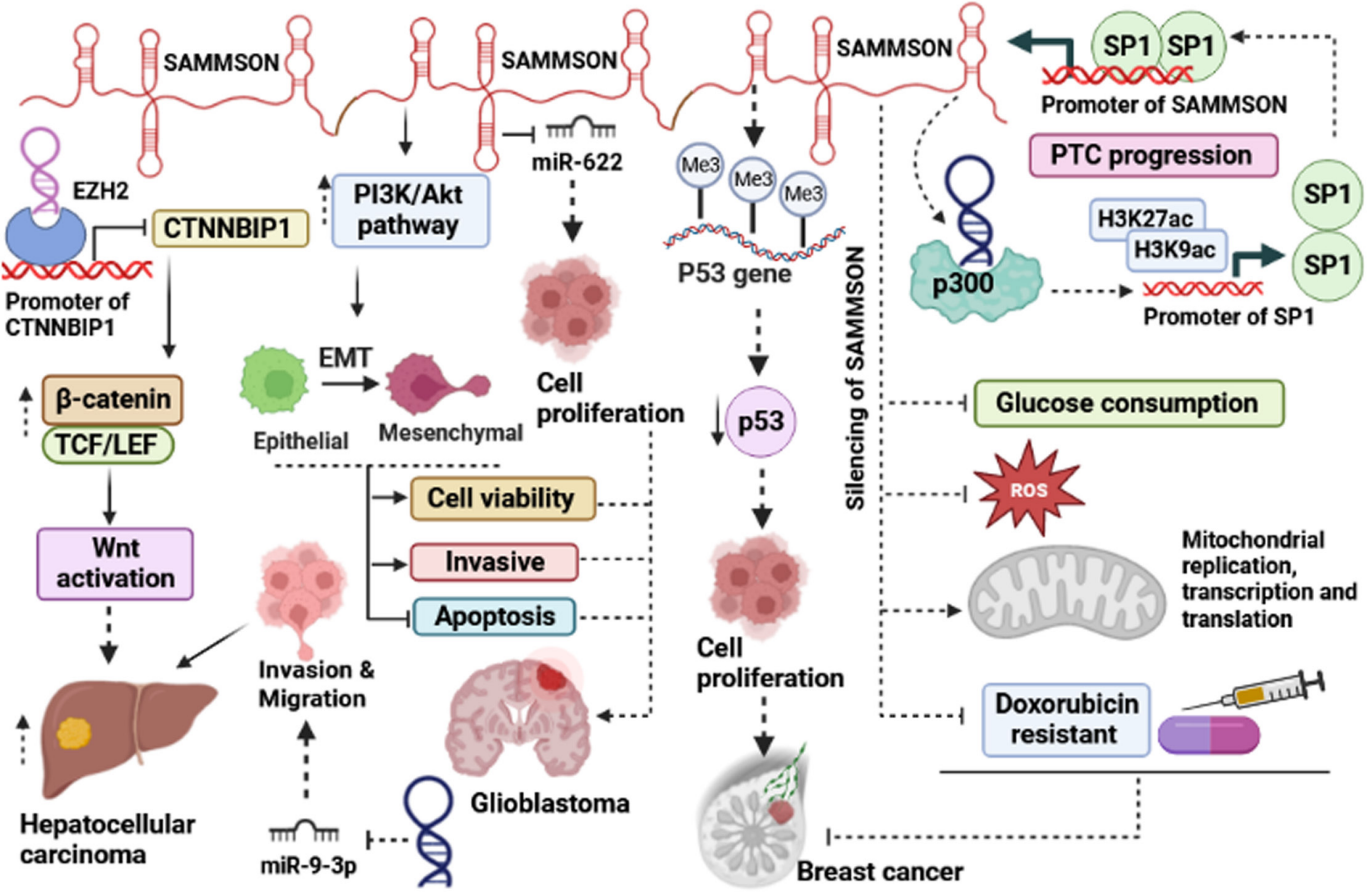
A schematic representation of the function of SAMMSON in different cancers. SAMMSON, by promoting Wnt activation, and sponging miR‐9‐3p increases hepatocellular carcinoma. In breast cancer, SAMMSON promotes tumour progression via facilitation of epigenetic modulations. SAMMSON, by sponging miRNAs affects different aspects of tumour behaviour. On the contrary, silencing of SAMMSON inhibits the production of ROS, and reduces drug resistance, which results in inhibiting the growth of cancer cells. In glioblastoma, SAMMSON via the activation of PI3K/Akt signalling pathway and sponging of miR‐622 effects EMT process and cell proliferation. Also in PTC, SAMMSON can interact with p300 and promotes the expression levels of SP1 as a transcription factor. TCF/LEF: T‐cell factor/lymphoid enhancer factor, EMT: Epithelial‐mesenchymal transition, Me: Methylation, SP1: A transcription factor, PTC: Papillary thyroid cancer, ROS: Reactive oxygen species.

Charlotte et al. reported that SAMMSON is involved in doxorubicin resistance in MCFdox (doxorubicin‐resistant) cell line (Figure 2). They reported that the expression levels of SAMMSON significantly were upregulated in MCFdox compared to the MCF‐7 cell line. Silencing of SAMMSON inhibits glucose consumption (decrease in the lactate/glucose ratio) while the rate of respiration remarkably increased by modulating complex I activity, and the levels of ROS production decreased. They reveal that after silencing of SMMSON, mitochondrial replication, transcription and translation increased.^11^ Xing et al. revealed that overexpression of SMMSON increased triple‐negative breast cancer (TNBC) cell proliferation. They reported that SMMSON exerts this function by facilitating P53 gene methylation.^23^

### 2.3 Gastric cancer

Past investigations revealed that the expression level of SAMMSON was upregulated in both patient samples and cancerous cell lines.^24^, ^31^ Sun et al. reported that the expression level of SAMMSON is closely related to distant metastasis, lymph node metastasis, and clinical stage, and patients with high expression of SAMMSON have a low overall survival time. Silencing of SAMMSON significantly reduces invasion and migration in gastric cancer cell lines.^24^ Amini et al. reported that SAMMSON expression has a positive association with tumour grade and can be considered as a potential diagnostic biomarker for tumours.^31^

### 2.4 Papillary thyroid carcinoma (PTC)

In vivo and in vitro investigations reveal that malignant phenotypes of PTC such as cell proliferation, invasion, metastasis and tumorigenicity be suppressed after SAMMSON silencing. As shown in Figure 2, the promoter of SAMMSON has 3 binding motifs for the Sp1 transcription factor. Upregulation of SP1 can promote SAMMSON expression at the transcriptional level by increasing the activity of its promoter. In the following, SAMMSON can bind to p300 (a histone acetyltransferase) to promote H3K27ac and H3K9ac levels on the promoter of Sp1, resulting in transcriptional activation of Sp1. The existing positive feedback loop Sp1/SAMMSON/ p300 has a pivotal role in PTC progression.^27^

### 2.5 Glioblastoma (GBM)

Past investigations have shown that the PI3K/Akt signalling pathway has a pivotal role in the progression of GBM.^32^ In GBM tissue and cancerous cell lines, the expression level of SAMMSON was aberrantly upregulated. Knockdown of SAMMSON remarkably reduced cell viability and invasive ability while promoting the induction of apoptosis. SAMMSON increases invasion by promoting the epithelial‐to‐mesenchymal transition (EMT) process. After the silencing of SAMMSON, the phosphorylation status of both PI3K (p‐PI3K) and Akt (p‐Akt) proteins significantly reduced that indicated SAMMSON was involved in the activation of this pathway (Figure 2).^12^ Xie et al. reported that SAMMSON inversely correlated with miR‐622. Overexpression of SAMMSON can promote cell proliferation by decreasing miR‐622 expression, while it had no significant effect on migration and invasion in GBM cancer cell lines (U87 and U‐373 MG).^26^

### 2.6 Hepatocellular carcinoma

In hepatocellular carcinoma, SAMMSON was upregulated and promotes invasion and migration; therefore, it has an oncogenic role. SAMMSON exerts this function by downregulating miR‐9‐3p expression (Figure 2). However, HCC cancer cell lines with an ectopic expression of miR‐9‐3p show less invasion and migration. Clinicopathological features do not affect SAMMSON expression, while between SAMMSON and miR‐9‐3p exists a negative correlation.^13^ Li et al. reported that SAMMSON was upregulated in liver tumour initiating cells (TICs) and liver cancer. The self‐renewal capacity of cancerous cells was impaired after silencing SAMMSON, and its ability was recovered after SAMMSON overexpression.^25^ EZH2 is a primary component of polycomb repressive complex 2 (PPRC2).^2^ CTNNBIP1 protein is a physiological negative regulator of the Wnt pathway that prevents interaction between CTNNB1 and TCF/LEF complex and inhibits Wnt signalling activation.^33^ SAMMSON through interaction with EZH2, suppresses the expression of CTNNBIP1. SAMMSON exerts this function by binding to the promoter of CTNNBIP1 and recruits EZH2. It can be concluded that SAMMSON promotes liver TIC self‐renewal and propagation of tumours via activation of the Wnt signalling pathway (Figure 2).^25^

### 2.7 Oral squamous cell carcinoma

The expression of SAMMSON in OSCC was found to be significantly higher in tissue samples, plasma samples, and OSCC cancer cell lines. Zheng et al. reveal that upregulation of SAMMSON is significantly associated with tumour differentiation, lymph node metastasis, TMN stage, and neighbouring tissue infiltration. They also indicated that the patients with high expression of SAMMSON had poor overall survival. Overall, the SAMMSON has a critical role in the progression of OSCC.^34^

## 3 SAMMSON LNCRNA AS A DIAGNOSTIC AND PROGNOSTIC BIOMARKER

Investigation of the function of SAMMSON as a diagnostic and prognostic value in different cancers has been the subject of a few studies. The expression of SAMMSON detected in different types of samples, including tissue, blood, plasma and cell lines; therefore, SAMMSON can be considered a non‐invasive potential diagnostic and prognostic biomarker in several types of cancers and a suitable therapeutic target for various tumours. Shao et al. *reported that the expression level of SAMMSON in plasma samples of PTC was upregulated and can be used* as a useful prognostic and diagnostic biomarker for PTC patients. Xie et al. demonstrated that SAMMSON expression increased in GBM plasma samples while not in diffuse neurosarcoidosis (ND) patients. They reported that SAMMSON has a diagnosis value for distinguishing GBM and ND. The ability of SAMMSON as a potential biomarker was evaluated in OSCC. Zheng et al reported that the expression levels of SAMMSON was be increased in plasma samples of patients with OSCC. They indicated that SAMMSON can serve as a novel diagnostic and prognostic biomarker in OSCC (Table 2).

**TABLE 2:** Summary of studies reported up‐regulation of SAMMSON in clinical samples.

Clinical samples	SAMMSON expression	Association with clinical data	Kaplan Meier	AUC	Refs
90 Paired tissues, 90 OSCC patient serum and 40 normal healthy controls	Up	Significant association with the clinical stages, Distant & Lymph node metastasis, Cancer invasion depth and Differentiation.	High SAMMSON expression significantly associated with poor overall survival and disease‐free survival	0.74 for OSCC serum samples vs healthy serum controls.	^34^
126 Paired tissues of GC	Up	Significant association with the clinical stages, distant metastasis and lymph node metastasis	High SAMMSON expression significantly associated with FS & OS	‐	^24^
70 Paired tissues and plasma of PTC	Up	Significant association with the clinical stages, Lymph node metastasis, and tumour size	High SAMMSON expression significantly associated with shorter overall and disease‐free survival time	0.91 for PTC plasma samples vs healthy controls	^27^
67 Malignant melanoma and 11 nevi tissues	Up	‐	Lower SAMMSON expression is associated with increased 10‐year OS	‐	^17^
40 Paired tissues of GC	Up	Significant association with the tumour grade & tumour size	‐	‐	^31^
56 GBM, and 35 control & 34 patients with DN	Up	‐	‐	0.88 for GBM vs control and 0.92 for GBM vs ND	^26^
68 TNBC Paired tissues	Up	Significant association with the clinical stages	‐	‐	^23^
70 Paired tissues of HCC	Up	‐	Patients with high levels of SAMMSON in HCC tissues had significantly lower overall rate	‐	^13^

Abbreviations: AUC, area under the curve; FS, Free survival; ND, neurosarcoidosis; OS, overall survival; OSCC, oral squamous cell carcinoma; TNBC, triple‐negative breast cancer; UP, upregulation.

## 4 CONCLUSION AND PERSPECTIVES

Generally, SAMMSON is remarkably upregulated in cancer; however Pan et al. reported that the expression level of SAMMSON was decreased in intracranial aneurysm.^14^ SAMMSON appears to have an impact on a variety of processes, including the regulation of mitochondrial function, cell proliferation, apoptosis, EMT, drug resistance, invasion and migration. LncRNAs are keys player in regulation of signalling pathways^35^, ^36^ and SAMMSON is involved in the regulation of several pathways such as Wnt, MAPK, PI3K, Akt, ERK and p53. SAMMSON can regulate various biological processes by affecting signalling pathways, so new strategies based on LncRNAs may be considered for treating specific cancers. Although previous research has revealed various aspects of SAMMSON's effects on tumour progression, many of the involved mechanisms could be the focus of future research. For instance, miRNAs and their target genes have a critical role in tumour development^37^, ^38^; therefore SAMMSON‐miRNA‐mRNA axis can be investigated in many tumours. SAMMSON can regulate some miRNAs (miR‐622 and miR‐9‐3p) and their target genes. In addition, single nucleotide polymorphism (SNPs) especially their SNPs that are located in 3p13–3p14 amplicon may effect SAMMSON expression, therefore in the next investigations exploring the association of SAMMSON expression with genetic variants is needed. LncRNAs are essential players in the process of gene transcription through epigenetic regulation. LncRNAs perform this function by regulating histone proteins or the methylation status of DNA.^39^, ^40^ SAMMSON via interaction with p300 (as a histone acetyltransferase) can increase H3K27ac and H3K9ac activity. Also, SAMMSON by modulating methylated histone H3 (H3K27me3) regulates FOXA2 expression. Previous research evidence has reported that lncRNA is linked to clinicopathological characteristics like lymph node metastasis, TMN stage, tumour grade and tumour differentiation. However, several studies have found that high SAMMSON expression is associated with poor overall survival. SAMMSON plays an important role in doxorubicin and vemurafenib resistance in breast cancer and melanoma, respectively. Several transcription factors, including Sp1 and SOX10, have been shown to bind upstream of the SAMMSON gene and increase its expression. A review of previous articles revealed that SAMMSON regulates cancer cell metabolism via its effects on rRNA maturation, protein synthesis, and mitochondrial function. SAMMSON promotes respiration, complex I activity, and mitochondrial capacity, all of which are required for cancer cell growth and proliferation. SAMMSON's pivotal role in cancer progression, taken together, makes it a potential therapeutic target for future research.

## AUTHOR CONTRIBUTIONS

**Majid Ghasemian:** Writing – original draft (lead). **Hossein Babaahmadi Rezaei:** Writing – review and editing (supporting). **Azam Khedri:** Writing – review and editing (supporting). **Chandrabose Selvaraj:** Writing – review and editing (equal).

## CONFLICT OF INTEREST STATEMENT

The authors declare that there is no declaration of interest to declare.
